# *TL1A* (*TNFSF15*) genotype affects the long‐term therapeutic outcomes of anti‐TNFα antibodies for Crohn's disease patients

**DOI:** 10.1002/jgh3.12398

**Published:** 2020-08-01

**Authors:** Katsuya Endo, Yoichi Kakuta, Rintaro Moroi, Katsutoshi Yamamoto, Hisashi Shiga, Masatake Kuroha, Takeo Naito, Yoshitaka Kinouchi, Atsushi Masamune

**Affiliations:** ^1^ Division of Gastroenterology, Department of Internal Medicine Tohoku University Graduate School of Medicine Sendai Japan; ^2^ Division of Gastroenterology Tohoku Medical and Pharmaceutical University School of Medicine Sendai Japan

**Keywords:** adalimumab, Crohn's disease, infliximab, *TL1A* (*TNFSF15*)

## Abstract

**Background and Aim:**

*TL1A* (*TNFSF15*) is a major Crohn's disease (CD) susceptibility gene, especially in the East Asian population, and is also known to be associated with some clinical phenotypes, such as stricturing and penetrating behavior. This study aims to investigate the association between *TL1A* genotype and the long‐term therapeutic outcomes of infliximab and adalimumab in Japanese CD patients.

**Methods:**

We investigated 119 biologic‐naïve CD patients treated with infliximab or adalimumab. *TL1A* ‐358C/T (rs6478109) was genotyped as a tag single nucleotide polymorphism (SNP) for CD risk or nonrisk haplotype of *TL1A* (the ‐358C allele is a risk allele for CD development). We compared the long‐term therapeutic outcomes of anti‐tumor necrosis factor (TNF) antibodies between the *TL1A* ‐358C/C group and the C/T+T/T group.

**Results:**

Sixty‐nine cases (58.0%) were homozygous for the risk allele (*TL1A* ‐358C/C group), and 50 cases (42.0%) were heterozygous for the risk allele or homozygous for the protective allele (*TL1A* ‐358C/T+T/T group). No significant differences were found in the cumulative retention rates and the relapse‐free survival between the *TL1A* genotypes. However, the surgery‐free survival was significantly lower in the *TL1A* ‐358C/C group than in the C/T+T/T group (log‐rank test, *P* < 0.05). Multivariate analysis showed that *TL1A* ‐358C/C was identified as an independent risk factor for surgery (hazard ratio, 4.67; 95% confidence interval, 1.39–22.1; *P* = 0.025).

**Conclusion:**

An association was found between the *TL1A* genotype and the therapeutic outcomes of anti‐TNF therapy. Our data indicate that the design of customized therapy with anti‐TNF antibodies using *TL1A* genomic information could be effective in the future.

## Graphical abstract

In this study, we investigated the association between *TL1A* genotype and the long‐term therapeutic outcomes of infliximab and adalimumab in Crohn's disease (CD) patients. We investigated 119 biologic‐naïve CD patients treated with infliximab or adalimumab and found an association between the TL1A genotype and surgery‐free survival during anti‐tumor necrosis factor (TNF) therapy. The patients whose genotypes are homozygous for the *TL1A* ‐358C (risk allele of CD development) show lower surgery‐free survival with anti‐TNF treatment.

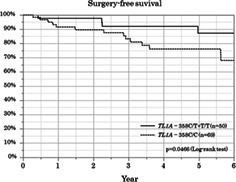

## Introduction

Crohn's disease (CD) is a chronic inflammatory bowel disease that involves the small and/or large intestine characterized by patchy transluminal inflammation, granuloma formation, and gut fibrosis. Quite a high number of CD patients experience complications during the disease course, such as gut stricture, fistula, perforation, abdominal abscess, and perianal fistula, which frequently require surgical treatments. The etiology of CD is still unclear, but the disorders of the gut mucosal immune systems caused by the various environmental factors and genetic factors are hypothesized as major pathogenesis. Concerning the genetic factors associated with the risk of CD development, various previous studies have identified the genes or loci, such as *NOD2/CARD15*,^1^ *TL1A* (*TNFSF15*),^2^ *ATG16L1*,^3^ *IL23R*,^4^ and *RAP1A*.^5^

TL1A is one of the important members of the tumor necrosis factor (TNF)/TNF receptor superfamily that play a critical role in various immunological responses involved in several inflammatory diseases, including CD.^6^, ^7^, ^8^ *TL1A* has been identified as a major CD susceptibility gene.^9^ The first report by Yamazaki *et al*. described highly significant associations of single nucleotide polymorphism (SNPs) and haplotypes within the *TL1A* genes in Japanese patients based on the result of a genome‐wide association study.^2^ Subsequent replication studies confirmed the similar significant association not only in Asian cohorts^10^, ^11^ but also in some Western cohorts.^12^, ^13^ *TL1A* has also been reported to be associated with some clinical phenotypes of CD. Kakuta *et al*. reported the association between *TL1A* SNPs and anal lesions.^10^ Hirano *et al*. reported the association between *TL1A* rs3810936 C allele and ileocecal location, structuring, and penetrating behavior.^14^ Yang *et al*. also reported that nonrisk allele homozygotes of some *TL1A* SNPs are risk factors for strictures/nonperianal penetrating complications and perianal fistula.^15^ Thus, *TL1A* has susceptibility not only to CD development but also to disease phenotypes. However, little is known about the association between *TL1A* genotypes and the long‐term prognosis of CD. In recent decades, anti‐TNFα antibodies, such as infliximab (IFX) and adalimumab (ADA), have been the main therapeutic agents for refractory CD patients. Hence, it is quite important to clarify whether the *TL1A* genotype affects the long‐term prognosis of anti‐TNF therapy for CD.

In this study, we investigated the association between *TL1A* genotype and the long‐term therapeutic outcomes of IFX and ADA in Japanese CD patients.

## Methods

### *Study design and patients*

This was a retrospective cohort study at a single center. We enrolled a total of 228 Japanese CD patients treated with IFX or ADA as a first biologic at Tohoku University Hospital between 2003 and 2013. Of 228 patients, we excluded patients who received anti‐TNF therapy as a postoperative maintenance therapy (*n* = 47), patients who could not be followed up after 8 weeks due to either primary nonresponse or intolerance (*n* = 8), and patients for whom genetic analyses were not available (*n* = 54). A total of 119 patients were investigated in this study.

This study was approved by the institutional ethics committee, and written informed consent was obtained from all patients.

### *Protocol of* *anti‐TNF* *therapy*

The anti‐TNF‐α antibodies were administered to CD patients with moderate to severe disease activities. IFX was administered at a 5 mg/kg infusion in weeks 0, 2, and 6 as an induction therapy. When a clinical response was observed, maintenance treatment with IFX (5 mg/kg every 8 weeks) was initiated. In some cases with IFX loss of response (LOR), IFX dose was increased to up to 10 mg/kg. ADA was injected subcutaneously at 160 mg/body in week 0 and 80 mg/body in week 2. When a clinical response was observed, 40 mg/body of ADA was administered subcutaneously every 2 weeks as maintenance. ADA dose escalation was not performed in the case of LOR because the dose escalation was not officially approved during our study period in Japan.

### *Genotyping of* *TL1A*

In this study, we performed the genotyping of *TL1A* ‐358C/T (rs6478109) as a tag SNP for CD risk or nonrisk haplotype. In a previous study, it was reported that ‐358C/T had the highest odds ratios for CD development^10^ and could be regarded as a tag SNP for CD risk or nonrisk haplotype.^2^ The ‐358C allele is a risk allele for CD development.

DNA was extracted from samples collected from the patients' peripheral blood. *TL1A* ‐358C/T (rs6478109) was genotyped using the TaqMan SNP Genotyping Assay Kit (assay ID: C_1305297_10) and the ABI StepOnePlus Real‐Time Polymerase Chain Reaction System (Applied Biosystems, Foster City, CA, USA) per manufacture's protocol.

### *Overall long‐term outcomes*

Kaplan–Meier methods were used to analyze the overall long‐term outcomes of anti‐TNF therapy by focusing on the following three end‐points: cumulative retention rate (end‐point: discontinuation of the agent due to any cause), cumulative relapse‐free survival (end‐point: clinical relapse), and cumulative surgery‐free survival (end‐point: surgical bowel resection). Clinical relapse was defined as the necessity of treatment stepup, including additional administration of steroids or thiopurine, dose escalation or switching of the anti‐TNF antibody, hospitalization, and surgery.

### *Comparisons of the long‐term outcomes among the* **TL1A** *genotypes*

We classified the patients into two groups based on the genotyping results: *TL1A* ‐358C/C group, which is homozygous for the CD risk allele, and *TL1A* ‐358C/T+T/T group, which is heterozygous or homozygous for the protective allele.

The log‐rank test was used to compare the long‐term outcomes between *TL1A* ‐358C/C group and C/T+T/T group. In addition, a multivariate analysis was conducted using the Cox proportional hazard model to assess whether the *TL1A* genotype could be an independent risk factor for poor long‐term outcomes.

### *Statistical analysis*

The Kaplan–Meier method was used for statistical analysis of long‐term outcomes. The log‐rank test was used to compare the long‐term outcomes between the two groups. Risk factors associated with the long‐term outcomes were examined using a Cox proportional hazard model. *P* < 0.05 was considered statistically significant for between‐group comparisons. JMP Pro 13.2.1 (SAS Institute Inc., Cary, NC, USA) was used for all statistical analyses.

## Results

### *Baseline characteristics of the patients*

Baseline characteristics of the patients are presented in Table 1. Patients included 77 males (64.7%). Thirty‐nine patients (32.8%) were diagnosed with CD at younger than 20 years of age. The disease duration was less than 3 years in 40 patients (33.6%) at anti‐TNF antibody induction. The disease locations were ileal, ileocolonic, and colonic in 16 (13.4%), 83 (69.7%), and 20 (16.8%) patients, respectively. The disease behaviors were inflammatory, stricture, and fistula in 38 (31.9%), 53 (44.5%), and 28 (23.5%) patients, respectively. Eighty‐six patients (72.3%) had anal lesion, and 74 (62.2%) patients had a history of intestinal resection. Smoking habit was confirmed in 39 (32.8%) patients; however, smoking history was unknown in 40 (33.6%) patients. Sixteen cases (13.4%) were treated with concomitant thiopurines. As for the genotyping results of *TL1A* ‐358C/T, the frequency of C allele, identified as the risk allele for CD, was 75.2%. Genotype frequency of *TL1A* ‐358C/T in the study population was C/C, C/T, and T/T in 69 (58.0%), 41 (34.5%), and 9 (7.5%) patients, respectively. Sixty‐nine cases (58.0%) were homozygous for the risk allele (*TL1A* ‐358C/C group), and 50 cases (42.0%) were heterozygous for the risk allele or homozygous for the protective allele (*TL1A* ‐358C/T+T/T group).

**Table 1:** Clinical backgrounds of the study population

	*n* (%)
Gender	
Male	77 (64.7)
Female	42 (35.3)
Age at diagnosis (year)	
<20	39 (32.8)
≧20	80 (67.2)
Disease duration at the biologics induction (year)	
<3	40 (33.6)
≧3	79 (66.4)
Disease location	
Ileal	16 (13.4)
Ileocolonic	83 (69.7)
Colonic	20 (16.8)
Disease behavior	
Inflammatory	38 (31.9)
Stricture	53 (44.5)
Fistula	28 (23.5)
Anal lesion	
No	33 (27.7)
Yes	86 (72.3)
Previous intestinal resection	
No	45 (37.8)
Yes	74 (62.2)
Smoking	
Yes	39 (32.8)
No	40 (33.6)
Unknown	40 (33.6)
Concomitant thiopurine	
No	103 (86.6)
Yes	16 (13.4)
Anti‐TNF antibody	
Infliximab	101 (84.9)
Adalimumab	18 (15.1)
Genotype frequency of *TL1A* ‐358C/T (rs6478109)	
CC	69 (58.0)
CT	41 (34.5)
TT	9 (7.5)
Frequency of *TL1A* ‐358 C/C group and C/T+T/T group	
*TL1A* ‐358C/C	69 (58.0)
*TL1A* ‐358C/T+T/T	50 (42.0)

TNF, tumor necrosis factor.

### *Overall long‐term outcomes of* *anti‐TNF* *therapy*

**Figure 1:**
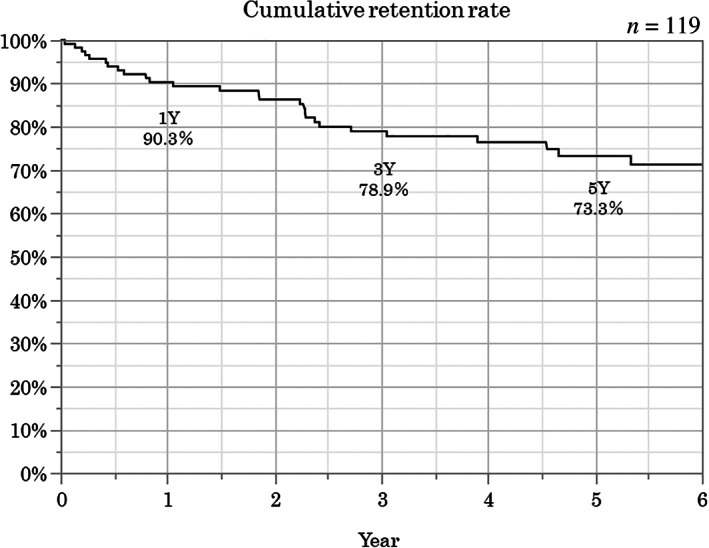
Overall cumulative retention rate. The cumulative retention rates at 1, 3, and 5 years were 90.3, 78.9, and 73.3%, respectively.

**Figure 2:**
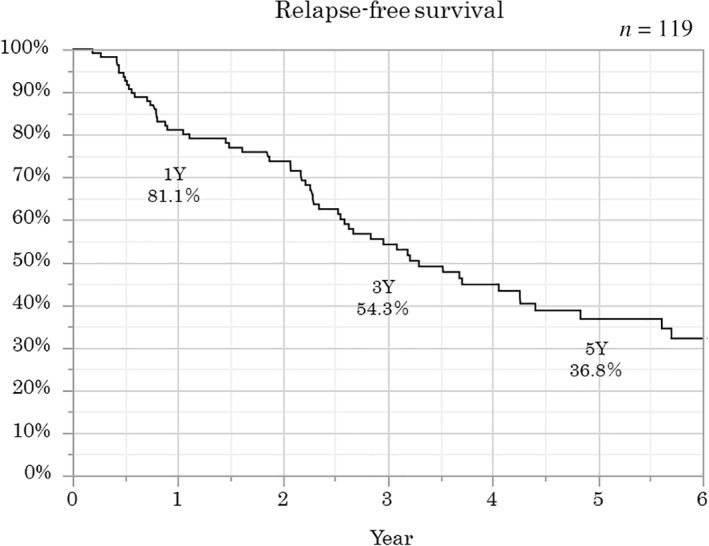
Overall relapse‐free survival. The relapse‐free survival rates at 1, 3, and 5 years were 81.1, 54.3, and 36.8%, respectively.

**Figure 3:**
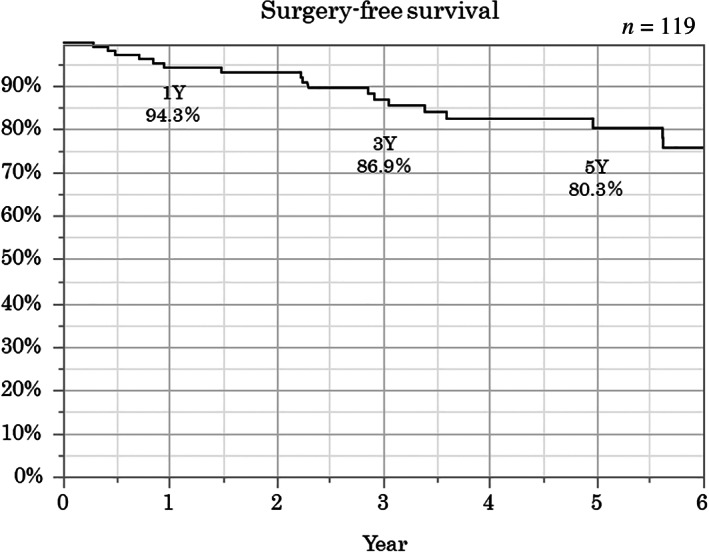
Overall surgery‐free survival. The surgery‐free survival rates at 1, 3, and 5 years were 94.3, 86.9, and 80.3%, respectively.

The Kaplan–Meier method was used to analyze the overall long‐term outcomes. The cumulative retention rates at 1, 3, and 5 years were 90.3, 78.9, and 73.3%, respectively (Fig. 1). The relapse‐free survival rates at 1, 3, and 5 years were 81.1, 54.3, and 36.8%, respectively (Fig. 2). The surgery‐free survival rates at 1, 3, and 5 years were 94.3, 86.9, and 80.3%, respectively (Fig. 3).

### *Impact of* **TL1A** *genotype for long‐term outcomes of* *anti‐TNF* *therapy*

**Figure 4:**
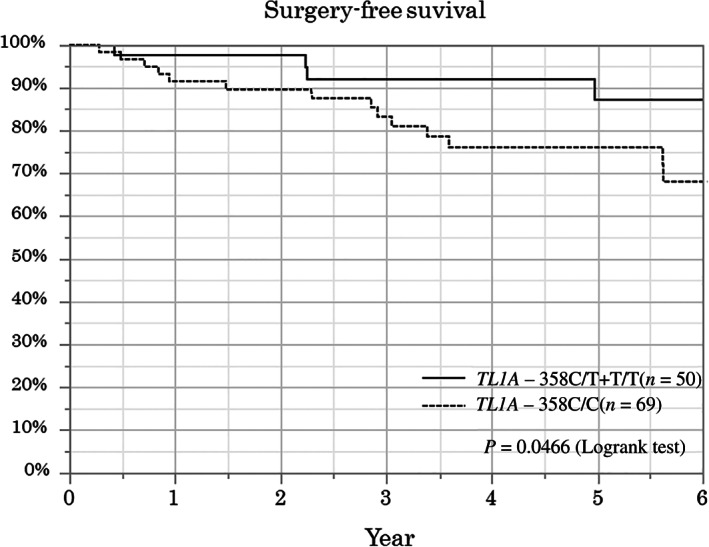
Comparison of surgery‐free survival between the *TL1A* ‐358C/C group and the C/T+T/T group. The surgery‐free survival rate was significantly lower in the *TL1A* ‐358C/C group than in the C/T+T/T group (log‐rank test, *P* < 0.05).

Associations between the clinical factors, including *TL1A* genotype, and the long‐term outcomes analyzed by univariate analysis (log‐rank test) are summarized in Table 2. The surgery‐free survival was significantly lower in the *TL1A* ‐358C/C group than in the C/T+T/T group (*P* < 0.05) (Fig. 4).

**Table 2:** Association between clinical factors and long‐term outcomes (univariate analysis†)

Clinical factor	*n*	Cumulative retention rate	Cumulative relapse‐free survival	Cumulative surgery free survival
		*P*‐value†		
Gender				
Male	77	0.1384	0.1604	0.8257
Female	42			
Age at diagnosis (year)				
<20	39	0.9087	0.4555	0.596
≧20	80			
Disease duration at the biologics induction (year)				
<3	40	0.9265	0.5584	0.733
≧3	79			
Disease location				
Ileal	16	0.2981	0.9937	0.3793
Ileocolonic	83			
Colonic	20			
Disease behavior				
Inflammatory	38	0.2231	0.9948	0.6217
Stricture	53			
Fistula	28			
Anal lesion				
No	33	0.3662	0.261	0.5588
Yes	86			
Previous intestinal resection				
No	45	0.9963	0.8593	0.4058
Yes	74			
Concomitant thiopurine				
No	103	0.087	0.2323	0.8989
Yes	16			
*TL1A* ‐358C/T genotype				
‐358C/T+T/T	50	0.6799	0.2082	**0.0466** ††
‐358C/C	69			

† Log‐rank test.

†† *P*‐value is less than 0.05.

Risk factors associated with the surgery‐free survival analyzed by multivariate analysis (Cox proportional hazard model) are presented in Table 3. No clinical factors were identified as a risk for surgery except for the *TL1A* genotype. *TL1A* ‐358C/C was identified as an independent risk factor for surgery (hazard ratio [HR], 4.67; 95% confidence interval [CI], 1.39–22.1; *P* = 0.025).

**Table 3:** Risk factors associated with the cumulative surgery‐free survivals (multivariate analysis†)

Risk factor	*n*	Cumulative surgery‐free survival	
		HR (95% CI)	*P*‐value
Gender			
Male	77	1	0.245
Female	42	1.96 (0.61–6.10)	
Age at diagnosis (year)			
<20	39	1	0.222
≧20	80	2.20 (0.64–8.37)	
Disease duration at the biologics induction (year)			
<3	40	1	0.7
≧3	79	0.806 (0.27–2.49)	
Disease location			
Ileal	16	1	0.308
Ileocolonic	83	0.467 (0.12–2.29)	
Colonic	20	0.202 (0.02–1.55)	
Disease behavior			
Inflammatory	38	1	0.881
Stricture	53	0.982 (0.22–4.18)	
Fistula	28	1.360 (0.24–7.91)	
Anal lesion			
No	33	1	0.329
Yes	86	0.571 (0.18–1.84)	
Previous intestinal resection			
No	45	1	0.874
Yes	74	1.119 (0.28–4.69)	
Concomitant thiopurine			
No	16	1	0.771
Yes	103	1.231 (0.25–4.48)	
*TL1A* ‐358C/T genotype			
‐358C/T+T/T	50	1	**0.025** ††
‐358C/C	69	4.674 (1.39–22.1)	

† Cox proportional hazard model.

CI, confidence interval; HR, hazard ratio.

†† *P*‐value is less than 0.05.

## Discussion

This study showed that the *TL1A* genotype is associated with surgery‐free survival during anti‐TNF therapy in Japanese CD patients. Patients whose genotypes are homozygous for *TL1A* ‐358C (risk allele of CD development) showed lower surgery‐free survival. To our knowledge, this is the first report to describe the association between *TL1A* genotype and the therapeutic outcomes of anti‐TNF therapy for CD.

The results of overall long‐term outcomes of anti‐TNF therapy in this study were almost similar to the previous study. Our cumulative retention rates of the agents seemed to be consistent with the previous reports investigating the Japanese CD cohort.^16^, ^17^ As for the result of relapse‐free survival, approximately 20, 45, and 65% of our patients relapsed at 1, 3, and 5 years, respectively. These results indicated that LOR to the biologics gradually increased year by year. These results are not inconsistent with the recent systematic review by Qiu *et al*., which showed that the annual risk for LOR in anti‐TNF treatment was 20.9% per patient‐year.^18^ The present outcome of surgery‐free survival seems to be satisfactory. Approximately more than 80% of the patients could avoid surgery at 5 years after anti‐TNF antibody inductions. Our results do not conflict with the recent observational cohort study reported by Eberhardson *et al*., which demonstrated that the cumulative rate of surgery exposed to TNF antibody at 5 years was 23%.^19^

The most important finding of this study is the association between *TL1A* genotypes and surgery‐free survival during anti‐TNF therapy. Some previous studies reported the associations between the *TL1A* genotype and the disease behavior of CD.^10^, ^14^, ^15^ However, there has been no report that describes the relationship between *TL1A* genotypes and the treatment outcomes of anti‐TNF agents. Basically, many studies have been performed on the role of TL1A on CD development. First, it was confirmed that increased TL1A expression was found in the inflamed gut or lamina propria T cells in CD patients.^20^, ^21^, ^22^ Second, some studies using TL1A overexpression murine models revealed that TL1A overexpression in lymphoid or myeloid cells exhibited enhanced intestinal fibrosis or intestinal strictures.^23^, ^24^ These findings indicate that TL1A can play an important role in promoting not only mucosal inflammation but also fibrostenosis in CD development. Concerning the relationship between *TL1A* genotypes and their expression, the increased TL1A expressions in stimulated T cells or monocytes from risk haplotype for CD were reported.^25^, ^26^ Furthermore, a recent study revealed that SNP rs4263839 in *TL1A* showed the strongest association with disease progression in the 10‐year follow‐up of CD patients with inflammatory phenotype at diagnosis.^27^ These previous findings lead to the hypothesis that patients homozygous for the *TL1A* risk haplotype could show higher TL1A expression, which could induce much severe disease progression and fibrostenosis than patients heterozygous or homozygous for nonrisk haplotype. Generally, the most common reasons for surgery are intestinal stenosis and fistula formation in the treatment course of CD. In this study, the most common reason for surgery was also intestinal stenosis. Thus, our results and previous evidence strongly suggest that the *TL1A* genotype affects the progression of the intestinal fibrostenosis even with anti‐TNF treatment.

According to aforementioned speculation, anti‐TL1A strategy could possibly be effective for the prevention of inflammation or fibrostenotic progression in CD treatment in the future. In fact, some recent in vivo studies have reported that the anti‐TL1A antibody reduces intestinal inflammation and fibrosis in murine colitis models.^28^, ^29^, ^30^ This basic evidence indicates that anti‐TL1A antibody could be effective for CD, especially for patients homozygous for the *TL1A* risk haplotype.

The limitations of this study include a small number of the patients from a single center, the retrospective study design, lack of genetic data in some patients, missing data on smoking habits, lack of endoscopic or cross‐sectional imaging evaluations, and the imbalance in the number of patients between IFX and ADA. Thus, further large cohort studies would be ideal to confirm the obvious association between *TL1A* genotype and the long‐term treatment outcomes in anti‐TNF antibody.

In conclusion, we found an association between the *TL1A* genotype and the therapeutic outcomes of anti‐TNF therapy for CD. The patients whose genotypes are homozygous for the *TL1A* ‐358C (risk allele of CD development) show lower surgery‐free survival with anti‐TNF treatment. Our data indicate that the design of customized therapy with anti‐TNF antibodies using *TL1A* genomic information could be effective in the future.
